# Unlocking the Therapeutic Potential of Adipose-Derived Stem Cell Secretome in Oral and Maxillofacial Medicine: A Composition-Based Perspective

**DOI:** 10.3390/biology13121016

**Published:** 2024-12-05

**Authors:** Chiara Giannasi, Francesca Cadelano, Elena Della Morte, Camilla Baserga, Camilla Mazzucato, Stefania Niada, Alessandro Baj

**Affiliations:** 1 Department of Biomedical, Surgical and Dental Sciences, University of Milan, 20100 Milan, Italy; francesca.cadelano@unimi.it (F.C.); alessandro.baj@unimi.it (A.B.); 2 IRCCS Istituto Ortopedico Galeazzi, 20157 Milan, Italy; elena.dellamorte@grupposandonato.it (E.D.M.); camillabaserga@gmail.com (C.B.); camilla.mazzucato@unimi.it (C.M.)

**Keywords:** adipose-derived stem cells, secretome, immunomodulation, regeneration, wound healing, maxillofacial medicine

## Abstract

The adipose-derived stem cell (ADSC) secretome is widely studied for its immunomodulatory and regenerative properties, yet its potential in maxillofacial medicine remains largely underexplored. This review takes a composition-driven approach, beginning with a list of chemokines, cytokines, receptors, and inflammatory and growth factors quantified in the ADSC secretome to infer its potential applications in this medical field. First, a review of the literature confirmed the presence of 107 bioactive factors in the secretome of ADSCs or other types of mesenchymal stem cells. This list was then analyzed using the Search Tool for Retrieval of Interacting Genes/Proteins (STRING) software, revealing 844 enriched biological processes. From these, key processes were categorized into three major clinical application areas: immunoregulation (73 factors), bone regeneration (13 factors), and wound healing and soft tissue regeneration (27 factors), with several factors relevant to more than one area. The most relevant molecules were discussed in the context of existing literature to explore their therapeutic potential based on available evidence. Among these, TGFB1, IL10, and CSF2 have been shown to modulate immune and inflammatory responses, while OPG, IL6, HGF, and TIMP1 contribute to bone regeneration and tissue repair. Although the ADSC secretome holds great promise in oral and maxillofacial medicine, further research is needed to optimize its application and validate its clinical efficacy.

## Simple Summary

The secretome of adipose-derived stem cells (ADSCs) holds significant promise for oral and maxillofacial medicine due to its rich composition of growth factors, cytokines, and other soluble or vesicle-embedded bioactive mediators that promote tissue regeneration and immunomodulation. Potential applications include enhancing wound healing, reducing inflammation, and stimulating the regeneration of hard and soft tissues. This could lead to improved outcomes in procedures such as bone grafting, soft tissue reconstruction, and the treatment of oral and facial defects. By harnessing the regenerative properties of ADSC secretome, clinicians may be able to achieve more effective tissue repair, ultimately benefiting patient recovery and quality of life.

## 1. Introduction

Mesenchymal stem cells (MSCs) are multipotent stromal cells that have gained significant attention in regenerative medicine due to their ability to modulate the immune system and orchestrate tissue regeneration through paracrine signaling, cell-cell contact, and differentiation [1,2]. Among the different harvesting sources, adipose tissue is considered particularly convenient thanks to its abundance, easy accessibility with minor discomfort at the donor site, and higher yield of MSCs compared to other tissues, such as bone marrow [3]. Moreover, it represents a common waste tissue from procedures such as aesthetic surgery (e.g., liposuction, buccal fat removal) and bariatric surgery follow-ups. In the last 5 years, 102 clinical trials have investigated the therapeutic potential of adipose-derived stem cells (ADSCs) against a variety of clinical conditions, encompassing arthritis and cartilage defects (*n* = 21), COVID-19 and respiratory diseases (*n* = 19), central nervous system disorders (*n* = 15), and diabetes-related conditions (*n* = 6). The full list of clinical trials is provided in Supplementary S1. Notably, most of these trials focus on orthopedic conditions, while none are related to oral and maxillofacial medicine. This is unexpected, since the clinical needs in these medical fields share several similarities, particularly in the areas of bone regeneration, trauma management, and control of surgical infections. Moreover, lipofilling and fat grafting, which benefit from the presence of ADSCs, are widely used techniques in maxillofacial surgery. While these common clinical procedures—such as Lipogems^®^, Cell-Assisted Lipotransfer, and Microfat and Nanofat injections—typically rely on cell transplantation, a groundbreaking study by Gnecchi et al., in 2006 was the first to demonstrate that the regenerative potential of MSCs is primarily driven by their paracrine effects rather than direct cell engraftment [4]. This finding shifted the focus of scientific research toward exploring the secretome as a promising alternative to traditional cell therapy. The MSC secretome comprises biologically functional factors of various types—such as cytokines, chemokines, growth factors, miRNAs, and bioactive lipids—released by the cells either in soluble form or encapsulated within extracellular vesicles (EVs). These factors have demonstrated therapeutic benefits comparable to cell transplantation while presenting fewer associated drawbacks. Despite well-documented safety across diverse clinical applications [5], MSC-based cell therapy still faces important challenges. Key obstacles include the heterogeneity of MSC populations, which can impact therapeutic efficacy, low engraftment rates requiring substantial cell expansion prior to administration, the risk of immunogenic side effects, and the time- and cost-intensive manufacturing and handling processes [6]. The advantages of a secretome-based, cell-free approach include lower manufacturing costs, easier storage, and the ability to cross biological barriers [7]. Over the last two decades, a growing body of preclinical evidence has supported the potential of the MSC secretome as an alternative to cell therapy in various medical fields [8], including immune and inflammatory diseases [9,10] and neurological conditions [11]. To obtain cell secretome, cells are cultured under specific conditions, and the conditioned medium containing the secreted factors is collected and processed for further analysis or application. The manufacturing process significantly influences the composition of the secretome, as numerous variables can affect both the quantity and quality of the secreted factors. These variables include donor-related features, harvesting source (e.g., adipose tissue, bone marrow, dental pulp), cell isolation technique, cell passage and confluence at the time of secretome production, medium composition, and any physical or biochemical conditioning. Indeed, all these parameters affect the secretory profile of cytokines, growth factors, miRNAs and EVs, which are crucial for the therapeutic efficacy of secretome in regenerative medicine. The starting point for this review is a list of factors previously quantified in the secretome of fully confluent ADSCs cultured for three days under serum-starvation (absence of Fetal Bovine Serum, FBS) [12]. This setting was chosen because adipose tissue is an accessible and convenient source of MSCs, and secretome production from ADSCs has been standardized and extensively characterized over the past decade [13,14,15,16]. First, the presence of the identified factors in the ADSC and/or MSC secretome was validated through a review of published studies. Then, factors were re-analyzed for the therapeutic potential in oral and maxillofacial medicine, a field where the application of ADSCs and their derivatives remains largely unexplored. Finally, the most promising effectors involved in the processes of immunomodulation, and regeneration of bone and soft tissue were selected and discussed in the context of the existing literature to explore their potentiality based on available evidence.

## 2. Composition of ADSC Secretome: Focus on Chemokines, Cytokines, Receptors, and Inflammatory and Growth Factors

The complete list of factors considered in this study (Supplementary S2) was quantified in ADSC secretome samples analyzed by protein array, as extensively described in [12]. After removing duplicates (genes encoding for multiple isoforms), a total of 107 gene IDs corresponding to the quantified proteins were identified. Initially, each factor was cross-referenced with published studies to verify its recurrent identification in the ADSC secretome, or more broadly in MSC one, to validate the reliability of the initial list before proceeding with further analysis (Table 1).

**Figure 1:**
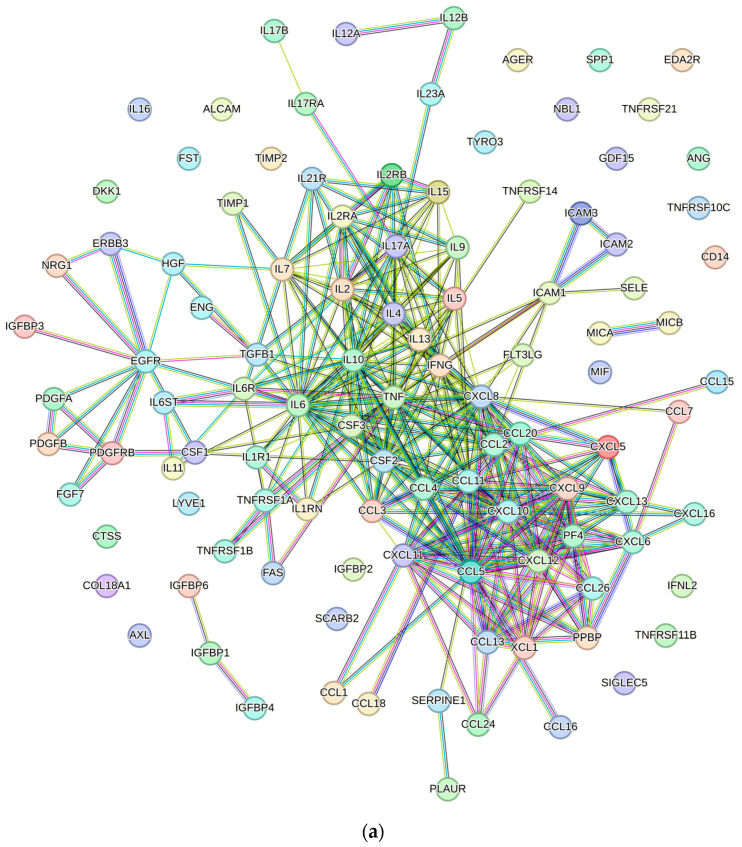

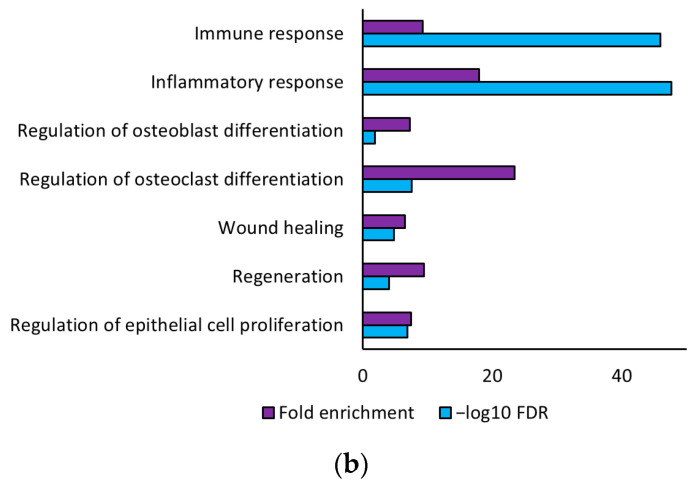
(**a**) Overview of the protein−protein interaction network of the 107 factors quantified in the ADSC secretome, generated using STRING software (version 12.0) with the interaction score threshold set to 0.900 for the highest confidence level. (**b**) Histogram showing the fold enrichment and false discovery rate (FDR) for seven selected pathways within the enriched biological processes highlighted by STRING analysis (Supplementary S3). Fold enrichment was calculated as follows: (number of observed proteins/number of proteins in the list)/(background gene count/number of protein-coding genes).

The list was then submitted to the Search Tool for Retrieval of Interacting Genes/Proteins (STRING) software (https://string-db.org/) to analyze protein–protein interactions and identify enriched biological processes (Figure 1a, Supplementary S3).

**Figure 2:**
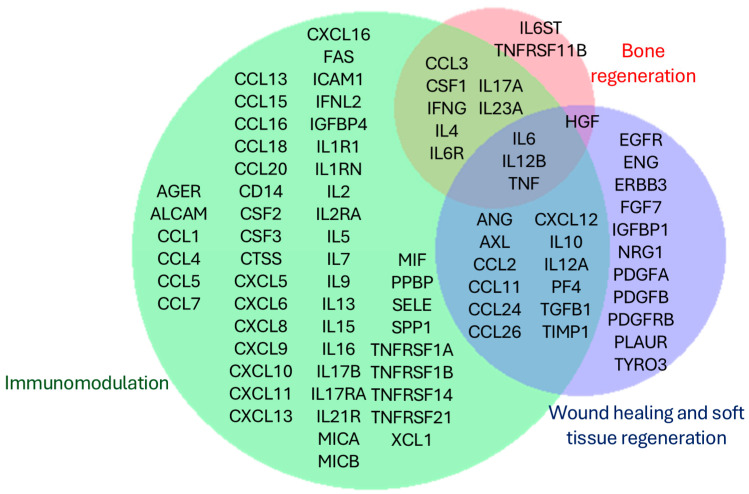
Venn diagram showing the unique and shared factors across the three major fields of ADSC secretome application in oral and maxillofacial medicine: immunomodulation, bone regeneration, wound healing, and soft tissue regeneration.

STRING analysis identified 844 enriched biological processes associated with the 107 input factors (Supplementary S3). Of these, seven were manually selected for their potential relevance to oral and maxillofacial medicine: immune response (GO:0006955), inflammatory response (GO:0006954), regulation of osteoblast differentiation (GO:0045667), regulation of osteoclast differentiation (GO:0045670), wound healing (GO:0042060), regeneration (GO:0031099), and regulation of epithelial cell proliferation (GO:0050678) (Figure 1b). These processes can be grouped into three primary application areas: (i) immunomodulation (GO:0006955 and GO:0006954), (ii) bone regeneration (GO:0045667 and GO:0045670), and (iii) wound healing and soft tissue regeneration (GO:0042060, GO:0031099, and GO:0050678) (Figure 2).

The following paragraphs will provide an overview of the factors present in the ADSC secretome that drive these effects, along with the clinical needs they may address in the field of oral and maxillofacial medicine. In each category, the discussion will also cover the role of specific microRNAs (miRNAs). These small, non-coding RNA molecules were first discovered by Prof. Victor Ambros and Prof. Gary Ruvkun [38], whose groundbreaking work earned them the Nobel Prize in Physiology or Medicine in 2024. miRNAs are key regulators of gene expression, and their presence in the ADSC secretome may significantly contribute to the hypothesized biological effects.

## 3. Immunomodulation

**Figure 3:**
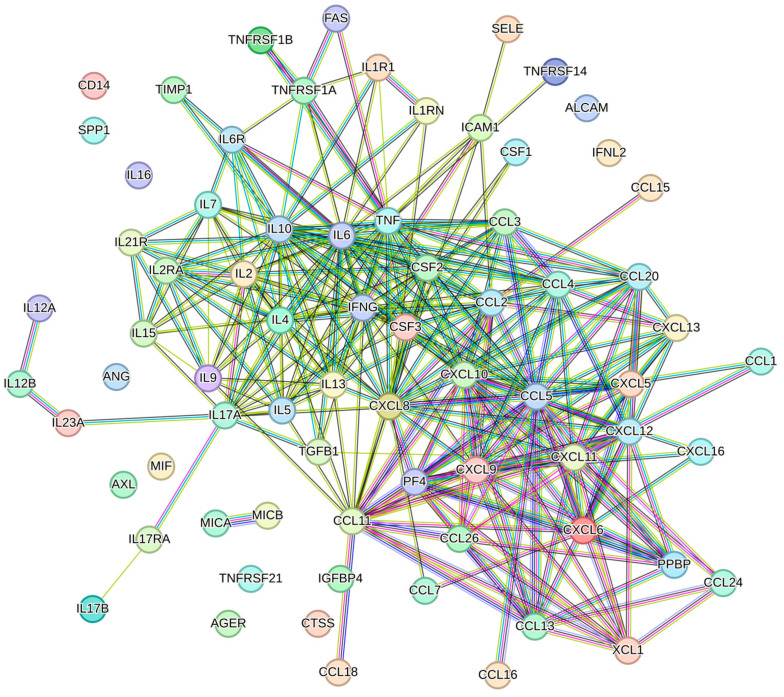
Protein–protein interaction network of the 73 factors associated with the pathways GO:0006955 (immune response) and GO:0006954 (inflammatory response). The network visualization was generated using STRING software (version 12.0, minimum required interaction score set to 0.900).

Biological products with immunomodulatory effects hold great potential in oral and maxillofacial medicine, addressing clinical needs such as periodontitis, stomatitis, temporomandibular joint osteoarthritis, and other inflammatory conditions. It is well-established that the MSC secretome exhibits key immunomodulatory properties [39]. In the presence of infection, it can enhance the antimicrobial response [40], while in inflammatory diseases characterized by immune overactivation, it can help suppress inflammation [10]. This dual capability, stemming from the wide array of immunomodulatory factors it contains, may be particularly advantageous in treating oral cavity diseases, where microbial infections and chronic inflammation often coexist. STRING analysis identified 73 factors linked to immune and inflammatory response (Figure 3), each influencing key immune effectors, including macrophages, neutrophils, and lymphocytes.

Macrophages are crucial players in the immune response and tissue healing, and they can be categorized into two main types based on their activation stimuli: M1 (classically activated) and M2 (alternatively activated) macrophages. Upon activation, M1 macrophages enhance antigen presentation, promote complement-mediated phagocytosis, and release pro-inflammatory cytokines and chemokines, facilitating the clearance of pathogens and initiating an immune response. Among the pro-inflammatory signals able to activate M1 macrophages, ADSC secretome contains Colony-Stimulating Factor 2 (CSF2), Tumor Necrosis Factor α (TNF), and Interferon γ (IFNG) [41], suggesting its role in promoting the initial stages of inflammation and microbial defense. IFNG plays a pivotal role in the early activation of macrophages. Upon binding to its receptor, it activates JAK1/2-mediated phosphorylation of STAT1, leading to the expression of pro-inflammatory genes. Additionally, IFNG signaling inhibits the STAT3 and STAT6 pathways, which are typically associated with M2 macrophage polarization, thereby reinforcing the M1 phenotype [42]. To sustain and amplify the M1 response, TNF and CSF2 are essential. TNF acts both as a ligand and as an autocrine signal, being produced in response to its own receptor activation, further promoting pro-inflammatory pathways. CSF2, upon binding to its receptor, triggers JAK2-dependent phosphorylation of STAT5, enhancing the production of inflammatory cytokines [43]. While M1 macrophages drive inflammation, M2 macrophages are involved in immune regulation, tissue remodeling, and wound healing. They can be further divided into three subtypes: M2a and M2b, which promote the Th2-mediated immune response, and M2c, which contributes to immune suppression and tissue regeneration. The ADSC secretome contains several cytokines that activate M2 macrophages, including the interleukins IL4, IL10, and IL13, Transforming Growth Factor β1 (TGFB1), and Colony-Stimulating Factor 1 (CSF1). These factors are crucial for M2 macrophage polarization and play a key role in facilitating tissue repair and resolving inflammation [44]. Specifically, IL4, IL13, and CSF1 promote M2a polarization through the JAK-STAT3/6 pathways, which facilitate cell growth and tissue repair. In contrast, TGFB1 signaling drives M2c polarization, a phenotype that plays a key role in the clearance of dead cells during the resolution phase of inflammation [45,46]. Neutrophils are short-lived yet essential cells of the innate immune system, acting as the body’s first line of defense against microbial infections. They contribute to microbial resistance primarily through phagocytosis, degranulation, and the release of bioactive mediators, including cytokines and reactive oxygen species. The protecting and activating effects of the MSC secretome on neutrophils have been recently demonstrated [47], highlighting the antimicrobial potential of this cell-free product. Key components in the ADSC secretome that enhance neutrophil function include TNF, Colony-Stimulating Factor 3 (CSF3), IFNG, Chemokine (C-X-C motif) Ligand 8 (CXCL8), and IL6 [47]. In detail, CSF3 and IL6 play crucial roles in neutrophil production during severe inflammatory conditions. They reduce the expression of CXCL12 and its receptor CXCR4, promoting the release of neutrophils from the bone marrow into the bloodstream and sustaining their proliferation through the JAK/STAT3 pathway [48]. TNF, IFNG, and CXCL8 facilitate neutrophil adhesion to the endothelial surface and promote diapedesis—the process by which neutrophils exit the bloodstream and migrate into target tissues. Once in the tissue, neutrophils engage in degranulation and NETosis, a defense mechanism where neutrophils release extracellular traps (NETs) composed of nucleic filaments that capture pathogens [48]. In addition to macrophages and neutrophils, the ADSC secretome plays a pivotal role in regulating lymphocytes, particularly T cells. Indeed, it contains numerous factors that influence T cell biology, including their activation, polarization, and apoptosis. Components such as hepatocyte growth factor (HGF), TGFB1, IL6, and IL10 help modulate the immune response by suppressing excessive inflammation and promoting immune balance [49,50,51]. These molecules often work in concert with other factors to produce varying outcomes. For example, TGFB1 and IL6, along with IL1 and IL23, drive differentiation toward helper T cells, which are crucial for neutrophil mobilization. In contrast, when TGFB1 combines with IL2 at the thymic level, it promotes the regulatory T pathway, supporting immune suppression. Additionally, IL6 and IL21 work together to foster long-term humoral immunity by promoting the differentiation of follicular helper T cells [52]. IL10 provides positive feedback by promoting the expansion of IL10-secreting regulatory T cells, which are critical for immune regulation in conditions like autoimmunity, chronic inflammation, and transplantation [53]. The ADSC secretome also contains a variety of miRNAs that play key roles in immunoregulatory and anti-inflammatory pathways [20]. In the context of temporomandibular joint osteoarthritis, the downregulation of miR-204 has been linked to an inflammatory phenotype [54], highlighting the therapeutic potential of the ADSC secretome as a source of this miRNA. Additionally, miR-146a has demonstrated anti-inflammatory and pro-osteogenic effects in experimental models of *Staphylococcus aureus.*-induced osteomyelitis, suggesting another promising therapeutic approach [55]. Similarly, miR-1260b has been shown to reduce alveolar bone loss associated with osteolytic inflammation in periodontitis [56]. Overall, given its rich composition and proven immunomodulatory effects, the ADSC secretome holds significant potential as a therapeutic option in maxillofacial treatments.

## 4. Bone Regeneration

**Figure 4:**
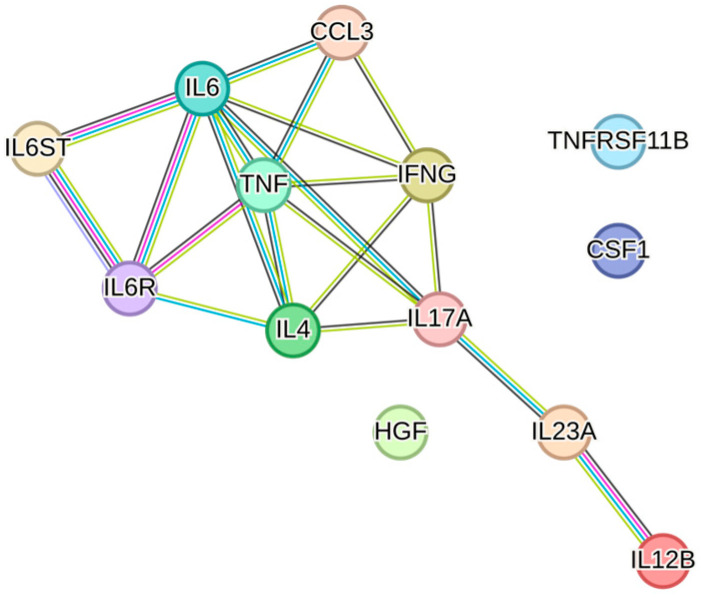
Protein–protein interaction network of the 13 factors associated with the pathways GO:0045667 (regulation of osteoblast differentiation) and GO:0045670 (regulation of osteoclast differentiation). The network visualization was generated using STRING software (version 12.0, minimum required interaction score set to 0.900).

Bone is a dynamic tissue that undergoes constant remodeling through the processes of resorption by osteoclasts and formation by osteoblasts. This turnover allows bones to adapt to stress, repair damage, and maintain their structural integrity. In maxillofacial medicine, effective bone regeneration is essential in cases of trauma, congenital defects, and following surgical procedures like implantology or tumor resection. The reconstruction of functional and aesthetic aspects affecting the head district is paramount for patient quality of life. Current strategies to achieve bone regeneration include traditional grafting techniques [57] and innovative tissue engineering approaches utilizing biomaterials, scaffolds, and MSCs [58]. In this context, using ADSC secretome in conjunction with biomaterials could offer a practical alternative to cell-based approaches. Two key biological pathways directly related to bone regeneration, identified through STRING analysis, are the regulation of osteoblast and osteoclast differentiation, which encompass a total of 13 associated factors (Figure 4).

Among these factors, Osteoprotegerin (OPG), also known as Tumor Necrosis Factor Receptor Superfamily Member 11B (TNFRSF11B), plays a vital role in maintaining bone homeostasis. As a key component of the RANKL/RANK/OPG system, it regulates bone regeneration by controlling osteoclast maturation and bone remodeling. It functions as a decoy receptor for RANKL and TRAIL, preventing their interaction with RANK receptors and thus inhibiting osteoclast-driven bone resorption. Mutations in the TNFRSF11B gene are linked to various conditions, including juvenile Paget’s disease [59], temporomandibular joint ankylosis [60], and alveolar bone loss associated with periodontal disease [61]. In these conditions, reduced OPG activity disrupts the balance between bone formation and resorption, leading to implant failure and peri-implant bone loss. Additionally, higher RANKL levels and lower OPG levels are associated with more severe periodontal disease, resulting in increased bone resorption [62]. Colony-Stimulating Factor 1 (CSF1) is another key component of the RANKL/RANK/OPG system. The CSF1/CSF1R signaling pathway promotes RANK expression, aiding in osteoclast formation and supporting the proliferation, survival, and differentiation of monocytes into osteoclasts [63]. CSF1 also plays a crucial role in conditions like osteoradionecrosis and osteomyelitis by recruiting macrophages and osteoclasts, both essential for bone healing and remodeling [64]. TNF is a pro-inflammatory cytokine that has a complex effect on bone regeneration, depending on its concentration and duration of exposure. During the bone healing and remodeling phase, TNF promotes osteoclast proliferation by increasing the production of the CSF1 receptor, aiding in bone regeneration [65]. At low concentrations (0.01–0.1 ng/mL), TNF enhances the expression of osteogenic transcription factors and bone marker genes through the induction of MAPK cascade, while at higher concentrations (10–100 ng/mL), it inhibits these processes via NF- κB pathway [66]. Hepatocyte Growth Factor (HGF) plays a crucial role in healing maxillofacial fractures by promoting the proliferation and differentiation of osteoblasts. By binding to the c-Met receptor, HGF activates the PI3K/Akt signaling pathway, which supports bone regeneration [67]. Additionally, HGF enhances fibroblast regenerative activity, initiating a signaling cascade in the early stages of wound healing that activates key pro-healing processes [68]. Additionally, HGF stimulates angiogenesis, ensuring an adequate supply of nutrients and oxygen to the healing bone, thereby accelerating the repair process [69]. In addition to their role as immune mediators, interleukins also influence bone regeneration. The IL23/IL17 axis contributes to T cell-mediated osteoclastogenesis by driving the proliferation of IL17-secreting T cell subsets in a positive feedback loop, while IL4 and IL13 share signaling pathways that inhibit bone resorption through STAT6-mediated OPG production [70]. In detail, IL4 suppresses osteoclast activity through multiple mechanisms: it directly inhibits their differentiation, lowers RANKL expression, increases OPG expression by osteoblasts, and reduces the number of inflammatory cells. Together, these actions reduce bone resorption, as observed in conditions like temporomandibular joint disorders [71,72,73]. In cases of maxillofacial fractures or bone diseases like osteomyelitis, IL4 may support healing by reducing inflammation and preventing excessive bone loss [74]. Recent studies have emphasized the role of miRNAs as key mediators in maxillofacial bone regeneration and remodeling [75]. Among those identified in the ADSC secretome, miR-21 is positively linked to bone matrix deposition and osteogenesis [76], while miR-29 inhibits bone resorption by modulating the RANKL/RANK/OPG system [20,77]. Together, all these factors interact within complex signaling networks that are crucial for effective bone regeneration, underscoring the potential of ADSC as a therapeutic strategy in this field.

## 5. Wound Healing and Soft Tissue Regeneration

**Figure 5:**
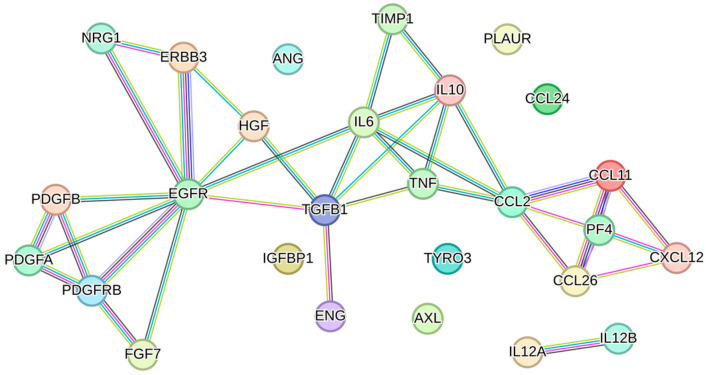
Protein–protein interaction network of the 27 factors associated with the pathways GO:0042060 (wound healing), GO:0031099 (regeneration), and GO:0050678 (regulation of epithelial cell proliferation). The network visualization was generated using STRING software (version 12.0, minimum required interaction score set to 0.900).

Wound healing and soft tissue regeneration are critical objectives in maxillofacial medicine due to the complex anatomy and vital functions of this region. These processes are essential not only for restoring functional integrity but also for maintaining the aesthetics of the orofacial area, which can be compromised by surgical procedures (e.g., dental implantology or periodontal surgery), trauma, or disease. Such conditions can impair key functions like speech and eating, ultimately diminishing the patient’s quality of life. Furthermore, promoting optimal healing can minimize scarring, enhance tissue integration, and reduce the risk of complications such as infections and implant failures. Wound healing and soft tissue regeneration involve a precisely regulated, spatiotemporally defined interplay of biological processes consisting of four overlapping stages: hemostasis, inflammation, cell proliferation (e.g., fibroblasts and keratinocytes), and extracellular matrix remodeling. Tissue engineering has emerged as a promising strategy to enhance these processes in the oral and maxillofacial areas. Innovative biomaterials and scaffolds such as hydrogels, nanofibers, films, and foam sponges are being explored for their biocompatibility and ability to promote soft tissue regeneration [78,79,80]. These materials are often combined with growth factors or stem cells to enhance their effectiveness. Recently, also cell-free approaches have been evaluated [81]. As tissue engineering efforts focus on innovative biomaterials like hydrogels, nanofibers, and foams, the incorporation of mesenchymal cell secretome offers a potent, cell-free approach to enhance tissue repair. In particular, ADSC secretome, by providing key bioactive molecules involved in tissue repair, can significantly enhance the biocompatibility and regenerative potential of scaffolds, complementing the effects traditionally achieved through direct use of stem cells. Of the 27 factors identified through STRING analysis (Figure 5), several play pivotal roles in the regenerative process of the orofacial district, as demonstrated in the literature.

Tissue inhibitors of metalloproteinases 1 (TIMP1) play a key role in regulating matrix metalloproteinase (MMP) activity, which is essential for extracellular matrix remodeling during wound healing. Like other members of the TIMP family, TIMP1 inhibits several MMPs with low selectivity by forming 1:1 non-covalent complexes. It is expressed by epithelial cells and fibroblasts near the wound edges, and its expression is closely associated with the healing process [82]. Transforming Growth Factor β1 (TGFB1) affects wound healing, being upregulated in response to acute injury. It promotes re-epithelialization, stimulates fibroblast proliferation, enhances collagen synthesis, and facilitates tissue remodeling through the activation of the SMAD2/3 pathway [83,84]. In vitro, it has been demonstrated that treatment with TGFB1 facilitates the commitment of primary human gingival fibroblasts to a myofibroblastic phenotype, a pivotal step occurring during tissue regeneration [85]. Neuregulin 1 (NRG1) promotes the proliferation and migration of keratinocytes and fibroblasts. Recently, similar effects were observed in vitro in human primary periodontal ligament stem cells, where NRG1 treatment also induces differentiation toward osteogenic and angiogenic lineages by combining with ERBB receptors and activating the corresponding signal transduction pathways [86]. Platelet-derived growth factor subunit B (PDGFB) is expressed during the early stages of gingival wound healing [87], and it enhances the proliferation of human gingival fibroblasts, overall promoting periodontal tissue regeneration [88]. Angiogenin (ANG) is a secreted protein that plays a key role in angiogenesis, a critical process for oral tissue repair and regeneration [89]. The application of recombinant human ANG, primarily in gel form, is currently being investigated with promising results as a tool to enhance wound healing in various models, including palate wounds, where it seems to promote epithelialization and reduce inflammation [89]. CXC motif chemokine 12 (CXCL12) acts as a chemoattractant for MSCs, guiding their recruitment to the injury site, where they orchestrate oral tissue regeneration [90,91]. Beyond these protein factors, various miRNAs are also involved in the processes of wound healing and soft tissue regeneration. Among them, miR-21 and miR-31 promote the migration and proliferation of fibroblasts and keratinocytes [92,93], while miR-29 influences matrix remodeling [94]. Additionally, miR-146 and miR-155 play crucial roles in the inflammatory phase of wound healing [95]. Notably, all these miRNAs have been identified in the secretome of both naïve and Interferon γ-primed ADSCs cultured under starving conditions (Supplementary Table S3 of [20]). Overall, the presence of these bioactive factors of different natures highlights the potential of ADSC secretome, alone or in combination with novel materials, as a biological tool able to improve wound healing outcomes and boost soft tissue regeneration in oral and maxillofacial medicine.

## 6. Preclinical Evidence of the Therapeutic Effects of MSC Secretome on Maxillofacial Conditions

The secretome from ADSCs and other MSC types has been extensively tested in preclinical models of maxillofacial disorders, including Temporomandibular Joint (TMJ), Osteoarthritis (OA), stomatitis, and periodontal disease. Recent studies have demonstrated that MSC secretome can improve various outcomes in TMJ OA models, such as enhancing morphological, histological, and molecular markers, reducing pain, promoting matrix synthesis, and modulating immune responses. These findings were highlighted in a systematic review by Jiang et al., [96]. Below are examples of the therapeutic effects described. The BM-MSC secretome has been shown to reduce inflammation and promote cartilage regeneration in a rabbit model of TMJ OA [97]. Similarly, in a murine model, the secretome from dental pulp MSCs reduced inflammation in both the cartilage and temporal muscle, also enhancing cartilage regeneration [98]. Notably, the ADSC secretome can be combined with other therapeutic approaches. El-Qashty et al., demonstrated that co-administration with low-level laser therapy restored joint structure, including normal cartilage and disc thickness, and significantly suppressed inflammation in a rat model of TMJ arthritis [99]. Moreover, the therapeutic potential of MSC secretome can be enhanced through cell priming. Liu et al., demonstrated that priming ADSCs with inflammatory cues (i.e., TNFα and IFNγ) amplified its therapeutic effects in a rabbit model of TMJ condylar osteochondral defects [100]. In a rat model of TMJ OA, the secretome from preconditioned dental pulp MSCs not only reduced inflammation but also promoted extracellular matrix production, supported subchondral bone repair, and mitigated joint degeneration [101]. Beyond TMJ disorders, the therapeutic potential of MSC secretome has also been explored in preclinical models of other oral cavity conditions, such as stomatitis and periodontitis. The immunomodulatory properties of MSCs have been studied in a large animal model of immune-mediated oral mucosal inflammation, feline chronic gingivostomatitis [102,103]. This condition resembles recurrent aphthous stomatitis in humans and shares features with other immune-mediated oral diseases, such as oral lichen planus, pemphigus, and pemphigoid. In this model, MSC treatment led to lasting improvement or complete remission in nearly 60% of treated animals. Given that the therapeutic effects of MSCs can be replicated by administering their secretome, this cell-free product should be considered a promising treatment option for such diseases. Periodontitis is a common dental condition characterized by inflammation of the tissues surrounding the teeth, often leading to the destruction of supporting structures and eventual tooth loss. Its multifactorial origin is primarily driven by bacterial biofilm on tooth surfaces, with disease progression largely influenced by the host immune response. Several studies have explored the effects of MSC secretome in periodontitis models, focusing mainly on the regeneration of the periodontal tissue [104,105,106,107] and bone [108]. Given the immunomodulatory properties of ADSC secretome, future research should also investigate its potential to modulate inflammation and control infection in periodontitis. Finally, the use of MSC secretome for reconstructing large craniofacial bone defects is strongly supported by numerous studies examining its therapeutic effects on calvarial bone defects (e.g., [109,110,111]).

## 7. Potential Clinical Applications in Maxillofacial Surgery

In light of these premises, the secretome shows significant clinical promise in maxillofacial surgery due to its bioactive components, including cytokines, growth factors, miRNAs, and EVs. These factors can enhance wound healing, bone regeneration, and immunomodulation, which are essential in oral and maxillofacial procedures. Potential applications include

- TMJ OA and joint disorders: The immunomodulatory properties of the secretome may aid in treating TMJ OA and other inflammatory joint disorders by reducing pain and inflammation and promoting cartilage repair. Intra-articular injections could improve outcomes for patients with chronic TMJ conditions.
- Bone regeneration: In dental implants and jaw reconstructions, the secretome can enhance bone regeneration. When used alongside bone graft materials, it may expedite osteogenesis and improve bone integration.
- Soft tissue reconstruction: The wound-healing agents in the secretome support soft tissue repair, which is beneficial for trauma or post-surgical recovery.
- Periodontal disease: The anti-inflammatory and antibacterial properties of the secretome could complement periodontal therapy, helping to reduce bacterial load and inflammation in infected gingival tissues.
- Postoperative pain and inflammation management: By modulating local inflammation and immune response, the ADSC secretome could be formulated into injectable or topical treatments to manage pain and swelling after maxillofacial surgery, potentially reducing dependence on conventional analgesics and anti-inflammatories.
- Fibrosis modulation: Since ADSCs possess the unique ability to modulate fibrosis [112,113,114], a secretome-based, cell-free alternative could be particularly beneficial for managing restricted mouth opening in patients with post-traumatic or post-surgical scarring, as well as in individuals with scleroderma.

## 8. Challenges and Future Perspectives

ADSC secretome has demonstrated significant promise in regenerative medicine, offering therapeutic potential comparable to cell therapies but with lower associated costs and easier handling and storage [115]. Cell-free therapeutics, such as EV-based products (e.g., ExoFlo™ and EV-Pure™), must undergo rigorous testing and comply with region-specific regulations. In the United States, the Food and Drug Administration (FDA) requires an Investigational New Drug (IND) application and a Biologics License Application (BLA) before commercialization, along with adherence to Good Manufacturing Practice (GMP) production protocols (https://www.fda.gov/drugs/how-drugs-are-developed-and-approved/types-applications (accessed on 11 October 2024)). In contrast, the European Medicines Agency (EMA) classifies cell-free therapeutics under the category of Advanced Therapy Medicinal Products (ATMPs). ATMPs must also comply with GMP standards, and depending on the application, some products may require a certificate of conformity to ensure they meet the European Medical Device Regulation (MDR) requirements (https://www.ema.europa.eu/en/human-regulatory-overview/advanced-therapy-medicinal-products-overview (accessed on 11/10/2024)). The variability in secretome composition—driven by factors such as cell source, culture conditions, the introduction of chemical or physical cues, cell passage, and post-collection variables—highlights the need for state-of-the-art, standardized protocols to ensure consistency and efficacy [116]. Quality control measures should include comprehensive characterization of the various components, such as proteins, miRNAs, and lipids, to ensure reproducibility and therapeutic potency. Regulatory guidelines must also address the manufacturing processes, including GMP compliance, and clarify the mechanism of action to establish safety and efficacy profiles for clinical use [117]. Standardized protocols and rigorous quality control can facilitate the regulatory approval and the clinical translation of secretome-based biotherapeutics.

## 9. Conclusions

In conclusion, this review highlights the significant potential of the ADSC secretome, characterized by its diverse composition of chemokines, cytokines, receptors, and inflammatory and growth factors, in advancing the management of several oral and maxillofacial conditions. The potent immunomodulatory properties, along with the ability to promote both bone and soft tissue regeneration, underscore its potential as an innovative biotherapeutic tool in this field. Harnessing the multifaceted capabilities of the ADSC secretome could lead to more effective and comprehensive treatments for complex clinical scenarios, providing novel solutions to medical challenges and ultimately enhancing patient outcomes.

## Figures and Tables

**Table 1:** Validation of the presence of the 107 factors in the secretome of ADSCs and other MSCs under different experimental conditions by comparison with the literature. Abbreviations: AF-MSCs = Amniotic Fluid MSCs; AM-MSCs = Amniotic Membrane MSCs; BM-MSCs = Bone Marrow MSCs; G-MSCs = Gingival MSCs; PL-MSC = Placental MSCs; UC-MSCs = Umbilical Cord MSCs; EIA = Enzyme Immunoassay; ELISA = Enzyme-Linked Immunosorbent Assay; LC-MS = Liquid Chromatography-Mass Spectrometry; LC-MS/MS = Liquid Chromatography–tandem Mass Spectrometry; WB = Western Blot.

Reference	MSC Type/s and Culture Conditions	Technique	Common Identified Factors
Riis et al., 2016 [17]	ADSCs 24 h Serum- and albumin-free medium Normoxia or hypoxia (1% O_2_)	LC-MS/MS	ALCAM, AXL, CSF1, IGFBP3, IGFBP4, IGFBP6, MIF, PLAUR, SERPINE1, TIMP1, TIMP2
Ritter et al., 2019 [18]	ADSCs 72 h Normoxia	ELISA	IL6, IL8, TNF
Bhang et al., 2014 [19]	ADSCs 48 h Serum-free medium Normoxia +/− 3D culture configuration	ELISA	CXCL12, HGF, VEGFA
Ragni et al., 2020 [20]	ADSCs 48 h Serum-free medium Normoxia +/− inflammatory priming	ELISA	ANG, AXL, CCL13, CCL2, CCL3, CCL4, CCL5, CCL7, CD14, CSF1, CTSS, CXCL10, CXCL12, CXCL16, CXCL5, CXCL8, CXCL9, DKK1, EGFR, ENG, FAS, FGF7, FST, GDF15, HGF, ICAM1, IFNG, IGFBP3, IGFBP4, IGFBP6, IL15, IL23A, IL2RA, IL2RB, IL6, IL6ST, MIF, OPG, PLAUR, SERPINE1, SIGLEC5, SPP1, TGFB1, TIMP1, TIMP2, TNFRSF1A, TNFRSF1B, TYRO3, VEGFA
Crisostomo et al., 2008 [21]	BM-MSCs 24 h Normoxia or hypoxia (1% O_2_) +/− inflammatory priming	ELISA	HGF, VEGFA
Chang et al., 2013 [22]	BM-MSCs 24 h Serum-free medium Normoxia or hypoxia (0.5% O_2_)	ELISA	HGF, VEGFA
Ryan et al., 2007 [23]	BM-MSCs 24 h Normoxia	EIA	HGF, IL10, TGFB1
Hodge et al., 2023 [24]	ADSCs 48 h Serum-free medium Normoxia +/− 3D culture configuration	Proteomic microarray	ANG, CCL2, CCL3, CSF2, CXCL16, CXCL8, ENG, FGF7, HGF, IGFBP1, IGFBP2, IGFBP3, NRG1, PDGFA, PDGFB, PF4, SERPINE1, TGFB1, TIMP1, VEGFA
Barone et al., 2023 [25]	ADSCs 48–72 h Serum-free medium Normoxia or hypoxia (2% O_2_) +/− 3D culture configuration	Cytokine array	AXL, CCL16, CCL3, CCL4, CSF3, CXCL11, CXCL5, CXCL8, EGFR, FAS, HGF, ICAM1, ICAM3, IGFBP3, IGFBP6, IL11, IL12A, IL12B, IL17A, IL1R1, IL2RA, IL6R, IL6ST, MIF, PLAUR, TIMP1, TIMP2, TNFRSF10C, TNFRSF11B, TNFRSF1A, TNFRSF1B, TYRO3, VEGFA
Hermann et al., 2023 [26]	ADSCs 24 h Serum-free medium Normoxia	ELISA	CCL2, CCL5, CSF1, EGFR, HGF, VEGFA
Linero et al., 2014 [27]	ADSCs 24 h Serum-free medium Hypoxia (2% O_2_)	Antibody array	ANG, CCL2, CCL5, CCL7, IL6, PDGFB, TGFB1, VEGFA
Blaber et al., 2012 [28]	ADSCs 72 h Normoxia	Multiplex immunoassays	CCL2, CCL3, CCL4, CCL5, CCL11, CSF2, CSF3, CXCL10, IFNG, IL1, IL10, IL12, IL13, IL15, IL17, IL1RN, IL2, IL4, IL5, IL6, IL7, IL8, IL9, PDGFB, TNF, VEGFA
Luo et al., 2014 [29]	AF-MSCs Normoxia	ELISA	IFNG, IL10, IL2, IL4, TGFB1
Liu et al., 2014 [30]	UC-MSCs 48 h Normoxia	ELISA	IL6, TGFB1, VEGFA
Tögel et al., 2007 [31]	BM-MSCs 24 h Serum-free medium Normoxia	ELISA	HGF, VEGFA
Hwang et al., 2009 [32]	BM-MSCs, PL-MSCs and UC-MSCs 72–96 h Normoxia	Cytokine array	CCL2, CXCL12, CXCL8, ICAM1, IL6, MIF, SERPINE1
Peshkova et al., 2023 [33]	ADSCs, BM-MSCs, G-MSCs, PL-MSCs and UC-MSCs 72 h Normoxia +/− 3D culture configuration	Luminex	CCL2, CCL24, CCL3, CCL4, CCL5, CCL7, CSF2, CSF3, CXCL10, FLT3LG, IFNG, IL10, IL12A, IL12B, IL13, IL15, IL17A, IL1A, IL1B, IL1RA, IL2, IL3, IL4, IL5, IL6, IL7, IL8, IL9, PDGF, TGF, TNF, VEGFA
Ragni et al., 2020 [34]	ADSCs 48 h Serum-free medium Normoxia Inflammatory priming	ELISA	AGER, ALCAM, ANG, AXL, CCL1, CCL11, CCL13, CCL15, CCL16, CCL18, CCL2, CCL20, CCL24, CCL26, CCL3, CCL4, CCL5, CCL7, CD14, CSF1, CSF2, CSF3, CTSS, CXCL10, CXCL11, CXCL12, CXCL12, CXCL13, CXCL16, CXCL5, CXCL6, CXCL8, CXCL9, DKK1, EDA2R, EGFR, ENG, FAS, FGF7, FLT3LG, FST, GDF15, HGF, ICAM1, ICAM2, ICAM3, IFNG, IFNL2, IGFBP1, IGFBP2, IGFBP3, IGFBP4, IGFBP6, IL10, IL11, IL12A, IL12B, IL13, IL15, IL16, IL17A, IL17B, IL17RA, IL1RN, IL2, IL21R, IL23A, IL2RA, IL2RB, IL4, IL5, IL6, IL6R, IL6ST, IL7, IL9, MICA, MICB, MIF, NBL1, NRG1, PDGFA, PDGFB, PDGFRB, PF4, PLAUR, PPBP, SCARB2, SELE, SERPINE1, SIGLEC5, SPP1, TGFB1, TGFB1, TIMP1, TIMP2, TNF, TNF, TNFRSF10C, TNFRSF11B, TNFRSF14, TNFRSF1A, TNFRSF1B, TNFRSF21, TYRO3, VEGFA, XCL1
Calligaris et al., 2024 [35]	AM-MSCs 48 h Serum-free medium Hypoxia (1% O_2_) +/− inflammatory priming	LC-MS	ALCAM, AXL, CCL2, CCL7, CSF1, CXCL5, CXCL6, CXCL8, DKK1, EGFR, ENG, FGF7, FST, GDF15, HGF, ICAM1, IGFBP3, IGFBP4, IGFBP6, IL11, IL6, IL6ST, MIF, NBL1, PDGFRB, PLAUR, SCARB2, SERPINE1, TGFB1, TIMP1, TIMP2, VEGFA
Uwazie et al., 2023 [36]	BM-MSCs 48 h Normoxia Inflammatory priming	Luminex	CCL11, CCL2, CCL3, CCL4, CCL5, CXCL10, CXCL8, CXCL9, FST, GSF2, GSF3, HGF, IFNG, IL10, IL12A, IL12B, IL13, IL15, IL17A, IL1RN, IL2, IL4, IL5, IL6, IL7, LYVE1, PDGFB, TNF, VEGF
Usategui-Martín et al., 2020 [37]	BM-MSCs 72 h Normoxia +/− co-culture	Protein microarray	ALCAM, ANG, AXL, CCL1, CCL10, CCL11, CCL13, CCL15, CCL16, CCL18, CCL2, CCL24, CCL26, CCL3, CCL4, CCL5, CCL7, CD14, CSF2, CSF3, CTSS, CXCL11, CXCL12, CXCL13, CXCL16, CXCL5, CXCL6, CXCL8, CXCL9, DKK1, EDA2R, EGFR, ENG, ERBB3, FAS, FGF7, FST, GDF15, HGF, ICAM1, ICAM2, ICAM3, IFNG, IFNL2, IGFBP1, IGFBP2, IGFBP3, IGFBP4, IGFBP6, IL10, IL11, IL12A, IL12B, IL13, IL15, IL16, IL17A, IL17B, IL17RA, IL1RN, IL2, IL23A, IL2RA, IL2RB, IL4, IL5, IL6, IL6R, IL7, IL9, LYVE1, MICA, MICB, MIF, NBL1, NRG1, PDGFA, PDGFB, PDGFRB, PF4, PLAUR, PPBP, RAGE, SCARB2, SELE, SERPINE1, SIGLEC5, SPP1, TGFB1, TIMP1, TIMP2, TNF, TNFRSF10C, TNFRSF11B, TNFRSF14, TNFRSF1A, TNFRSF1B, TNFRSF21, TYRO3, VEGFA, XCL1

## Data Availability

Not applicable.

## Supplementary Materials

The following supporting information can be downloaded at https://www.mdpi.com/article/10.3390/biology13121016/s1, Supplementary S1: List of clinical trials retrieved from a search conducted on 21 August 2024, on the ClinicalTrials.gov website using the keyword ‘adipose mesenchymal cells,’ with the filter ‘interventional’ for study type, and a study start date of 1 January 2020. The search initially identified 107 clinical trials, which were manually reviewed, resulting in a final count of 102 trials; Supplementary S2: Complete list of factors quantified by protein array in three out of three ADSC secretome samples, as reported in Giannasi [12]. Column A contains the original list, with the mean concentration expressed in pg/mL. Column B lists the corresponding gene IDs submitted to STRING. Duplicates are highlighted in red; Supplementary S3: List of enriched biological processes obtained through STRING analysis. Pathways selected for anti-inflammatory and immunomodulatory effects are highlighted in green, those related to bone regeneration are highlighted in red, and pathways associated with wound healing and soft tissue regeneration are highlighted in blue.
